# Rheumatism

**Published:** 1906-05-05

**Authors:** 


### Progress in Medicine and Surgery.

### RHEUMATISM.

(*Continued from page* 67.)

**The Forms of Rheumatoid Arthritis.**—The process of separating and classifying the various diseases once described as rheumatism has gone on rapidly during the last 50 years, and now a general feeling has arisen that even what is known as rheumatoid arthritis or arthritis deformans really comprises several distinct disorders. Further study too has shown the close analogy which some of its forms bear to the joint diseases which follow infectious diseases such as dysentery and gonorrhœa, so that it becomes extremely hard to decide upon any practical mode of classification. The difficulty is enhanced by the uncertainty of the bacteriology, by the rarity of post-mortems during the acute stages, and the change of symptoms presented as we pass in a single case from an acute to a chronic stage. Indeed, as Llewellyn Jones remarks, there may be diverse changes in different joints at the same time in one patient. In the discussion at the Medical Society in November, Garrod confessed that no single morbid entity corresponded to the rheumatoid arthritis of the text-books, but personally he would confine the name to the systemic disease with fusiform joint swellings and no osteoarthritic changes during the acute stage. He also suggested that spondylitis, malum coxæ senile, and similar affections do not arise from any single specific cause. Cave thought the line should be drawn between trophoneuroses and direct infections of joints. In some of the latter cases the micrococcus rheumaticus had been found in carious teeth, and probably similar sources of infection were discoverable in all. Bertram Abrahams thought that the division of the acute and chronic forms of arthritis had gone too far, and that it was merely a question of progress whether we had only synovial changes or others in the bones and cartilages; while Poynton took the ultra conservative view of a common origin for acute rheumatism and arthritis deformans based on the reproduction of arthritic lesions after the injection into animals of his micrococcus. It might be thought that Hale White's type of synovial changes in young females was a fairly definite group, but Robinson shows that some cases are probably due to a streptococcal infection, since rapid improvement has been noted after the injection of antistreptococcal serum. The arthritis of dysentery is said by Sandwith to be followed by joint changes like those seen in common forms of rheumatoid arthritis; but Beveridge, at Pretoria, where it occurred in 3 per cent. of the cases of dysentery, found that only a slight stiffness remained after the disappearance of the effusion. In America a crowd of competing systems of classification have recently appeared, none of which have as yet been generally accepted. We are struck, however, with the prominence given by American writers to a special type of joint disease known as Schüller's villous arthritis, due to a chronic polyarticular infection, and marked by permanent changes in the periarticular structures, without lesions of the bone or cartilage. Schüller found a definite organism present in all his cases; but Fayerweather, after taking most elaborate precautions, obtained totally different ones, and claimed that the English cases of rheumatoid arthritis, in which Bannatyne obtained still a third type of organism, were nevertheless clinically identical. He is therefore driven to the belief that all these organisms were merely secondary invaders. Another remarkable suggestion by P. W. Nathan is that some cases of osteoarthritis in the aged are due to arterio-sclerosis in the vessels of the part attacked. He finds that whilst there are no inflammatory changes, atrophic ones take place in the ligaments and synovial membranes with fibrillation of the cartilages and general deformity. The patients, too, suffer from cold hands and feet, and have a slow, hard pulse. It will be interesting to see whether observers on this side of the water can confirm this view. Certainly no explanation of the condition has been given beyond the vague phrases which ascribe it to "degenerative changes." One of the most popular classifications at the present time is Goldthwaits', but it is hard to see that any great advance is given by it. Indeed in the existing state of our knowledge no scientific classification seems possible. His five groups are (1) villous arthritis, a non-progressive disease, which he finds typically present in the knee with creaking and tenderness. (2) Atrophic arthritis, a progressive disease with distortion, swelling, and absorption of the cartilages. The fingers are affected early, and the jaw is often involved. (3) Hypertrophic arthritis with thickened cartilages, outgrowths, and increased density of the bones. The vertebræ may be fused together. (4) Infective forms, such as those due to typhoid, dysentery, and the gonococcus, to pneumonia or the organisms which complicate tuberculosis. (5) Chronic gout, which he thinks is slowly progressive, and should be separated from the acute form. It is marked by the deposition of urate of soda near the joints and the absorption of bone. It is difficult to see where osteoarthritis of the hip would come in, and there is after all great probability that his atrophic and hypertrophic types are really infectious, whether sequelæ of a tuberculous or some other poison. We need a classification based throughout on one principle, either on the causes of each type or on the clinical symptoms. The former is at present impracticable, for we do not know them, and are obliged to ascribe many cases to such negations as "autointoxications," "uric acid," and "intestinal toxins." A careful grouping according to clinical symptoms may finally show how the affections of known origin such as dysenteric joints compare with others, and this may in turn enable us to say that such and such types are of infectious origin, though the exciting cause may have passed away, or be situated at some distance from the affected joint. It would be most valuable to find some test or group of symptoms by which we could mark off all cases of infectious origin, all those attacks of definite course but self-limited duration which follow upon an invasion from without. Such a distinguishing mark should not be unattainable, even if we cannot isolate the *causa morbi.*

